# Mechanical Analysis of HPFRCC Precast Composite Column

**DOI:** 10.3390/ma18071567

**Published:** 2025-03-30

**Authors:** Tingting Lu, Bin Wang, Haowei Jin, Yuxiang Wen

**Affiliations:** Shaanxi Key Laboratory of Safety and Durability of Concrete Structures, Xijing University, Xi’an 710123, China; b635103208@163.com (B.W.); jhw523@163.com (H.J.); lx549499@163.com (Y.W.)

**Keywords:** prefabricated composite column, HPFRCC material, axial compression performance, bearing capacity calculation model

## Abstract

In order to improve the physical and mechanical properties and the ability to perform in practical applications of prefabricated monolithic composite columns, high-performance fiber-reinforced cementitious composites (HPFRCC) material was prefabricated into mold shells to form HPFRCC precast monolithic composite columns. Through the axial compression test, the axial compression failure form, failure mechanism, bearing capacity, deformation ability, and influencing factors were studied. The results showed that compared with RC precast monolithic composite column, the HPFRCC specimens showed better deformation performance. HPFRCC prefabricated shells provided additional restraint beyond stirrups. The HPFRCC composite columns’ yield compressive strain increased by 11.59% on average compared with the RC composite column, and the peak compressive strain increased by 10.92%. The larger the *ρ*_v_ of stirrups was, the larger the compressive strain of the key point of the columns was. Compared with the FC-P-01 (*ρ*_v_ was 1.05%), the yield compressive strain of FC-P-02 (*ρ*_v_ was 1.48%) increased by 21.63%, and the yield compressive strain of FC-P-03 (*ρ*_v_ was 0.74%) decreased by 11.20%. The calculation model of the axial bearing capacity of the HPFRCC composite column was established through theoretical mechanical analysis, and the calculated values of the model fit with the experimental values.

## 1. Introduction

The prefabricated concrete structure is a building form with the advantages of efficient construction, low emission, and low energy consumption. Due to the large size and self-weight of the completely prefabricated component, the cost of hoisting and transportation is higher, and the integrity of the completely prefabricated structure decreases. The prefabricated monolithic structures with the concept of “integral” promote the rapid development and application of the precast structure, and the mechanical properties have been widely studied [1,2].

To enhance the physical and mechanical properties of the precast composite members, researchers applied various new materials to the composite members to study their mechanical performances [3,4]. HPFRCC has the properties of tensile strain hardening and micro-crack development. Research showed that HPFRCC effectively improved the shear resistance and deformation ability of the core area of the joint, and enhanced the ability of bearing, deformation, and damage resistance of the structure when applied to the expected damage site [5,6]. In order to achieve further improvement in the mechanical behavior of the composite members, researchers used HPFRCC in the composite members to enhance the mechanical behavior of the prefabricated structure. Zhou Q. et al. [7] used ECC precast formwork and post-cast concrete to form components and studied the bonding properties through the tensile test. The test showed that the tensile strength of the bonded surface of ECC precast formwork reached 87.5% of its tensile strength, which met the tensile demand of composite components using precast formwork. Some researchers [8,9] prefabricated HPFRCC materials into formwork to form assemblies with concrete components. The results showed that the HPFRCC prefabricated formwork improved its bearing capacity and stiffness, in which case the ability to bear can be significantly improved by increasing the thickness of the prefabricated shell. Fiber-woven mesh could delay the cracking of the prefabricated shell and limit the development of cracks. Using UHPC formwork instead of traditional formwork as the permanent formwork of components was helpful in improving the overall working performance of the composite beam [10]. The cracking load, peak load, and stiffness of the composite beam at yield were significantly improved, and the peak load was increased by about 10%. Pan Z.F. et al. [11] conducted a study on the seismic performance of new reinforced engineering cement-based composite (RECC/C) columns. The research concluded that RECC/C significantly improved the shear capacity, energy dissipation capacity, and ductility of columns compared with RC; the increase in stirrup and shear span ratio enhanced the ductility and energy-consuming capacity of columns.

The above studies showed that the use of HPFRCC materials as permanent templates for prefabricated structures or composite members could improve the mechanical properties of assembled monolithic members. In order to further improve the mechanical performance of the composite members and the integrity of the prefabricated structure, welded steel mesh was cast in a prefabricated HPFRCC material shell to compose a new HPFRCC prefabricated shell structure system. And a new type of reinforced HPFRCC monolithic composite column was formed with the post-poured concrete. Through the axial pressure performance test, the failure state, failure mechanism, and influencing factors were analyzed. Then, the bearing capacity calculation model will be established through the theoretical mechanics analysis. The research results will provide a theoretical basis for improving the mechanical performance of prefabricated structures and promoting their engineering applications.

## 2. Materials and Methods

### 2.1. Experimental Design

**Figure 1:**
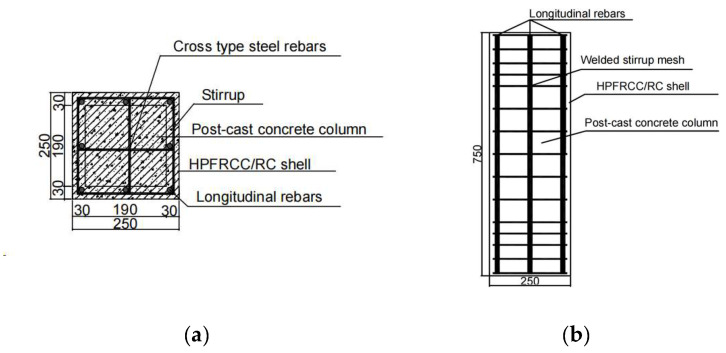
Dimensions diagram of specimens (units in mm). (**a**) Schematic view of the cross-section. (**b**) Schematic view of the section.

To study the failure process, bearing capacity, and ductility of HPFRCC columns, nine composite columns were designed based on the Code for Design of Concrete Structures [12]. The consideration factors were the material of the prefabricated shell, the reinforcement ratio of stirrups (*ρ*_v_), and the longitudinal reinforcement ratio (*ρ_t_*). The specimen numbers were FC-P-01~FC-P-05, RC-P-01, RC-P-02, RC-P-03, and RC-C-1. RC-C-1 was a cast-in-place column, and the rest were composed of two parts: reinforced precast shell and post-cast core concrete. Specimens FC-P-01~FC-P-05 were composed of the HPFRCC prefabricated shell with strength grade FC40. And the prefabricated shells of RC-P-01~RC-P-03 were made of C40 classic concrete. All the post-cast concrete and the concrete material of RC-C-1 were made of C40 commercial concrete. The cross-section size of the specimens was 250 mm × 250 mm, the height of the specimens was 750 mm, and the thickness of the prefabricated shell was 30 mm. The dimensions and parameters of the specimens are shown in Figure 1 and Table 1.

During the making process of the composite columns, the reinforced bar meshes were welded first. Then, the reinforced bar meshes were embedded in the prefabricated shells when the shell was cast. Finally, concrete was poured to form the prefabricated monolithic composite column. At the same time, cube blocks, prismatic blocks, and tensile test pieces were made for the material mechanical properties. The blocks were poured and cured under the same conditions as the specimens.

### 2.2. Mechanical Properties of Materials

The HPFRCC materials used in this test are as shown in Table 2, and PVA-imported fiber materials produced by Kuraray in Japan were adopted. The model was KURALINK-II-12 mm PVA fiber, and the fiber properties are shown in Table 3. Cement used was P·O 42.5 Portland cement; fly ash was sourced from Henan Hengyuan New Material Co., Ltd., Xingyang, China, Quartz sand was in the range of 16~120 mesh; and the water reducer used was a polycarboxylic acid high-performance water reducer. Commercial concrete was used to cast the precast shell of the RC composite column and the core concrete of each specimen. The strength grade of HPFRCC material was FC40, the ordinary concrete strength was C40, and the compressive strength of HPFRCC material and concrete is shown in Table 4. HPFRCC material tensile strength was measured by dumbbell-type test blocks, according to the corresponding specification, using an axial tensile test. The average value was 5.2 MPa. The tensile strength of concrete was calculated according to the axial compressive strength of concrete. In this paper, the HRB400 grade rebar was selected as the longitudinal rebar, and the HPB300 grade rebar was used as the stirrup. The mechanical properties of rebar are listed in Table 5.

### 2.3. Test Equipment and Loading

**Figure 2:**
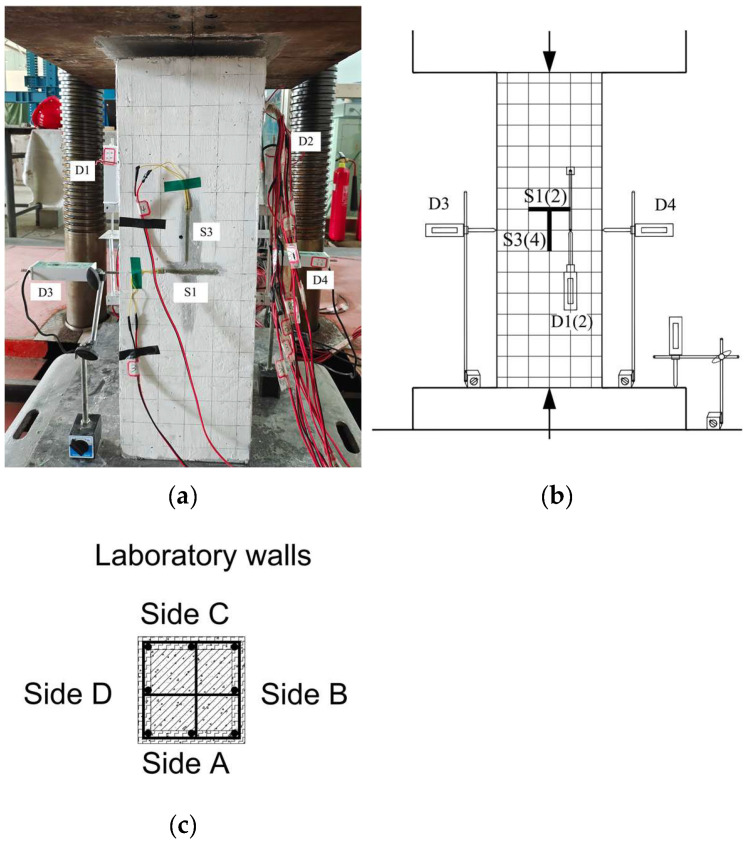
Test layout details. (**a**) Loading test. (**b**) Schematic diagram of test loading device. (**c**) Column specimen location.

The axial compression test was conducted on an electro-hydraulic servo compression shear test machine with a maximum loading load of 5000 kN. To facilitate the description of the test process, we named the four faces of the column specimens A, B, C, and D according to their positions in the laboratory. The strain of the steel bar and concrete during the test was measured by the data obtained from the resistance strain gauge. The displacement meters D1 and D2 were arranged in the middle of the A and C faces of the column specimen to measure the strain in the axial direction of the columns during the test. In addition to that, two displacement meters (D3, D4) were arranged in the middle of the B and D faces of the column specimen in a symmetrical way to measure the strain of the columns in a horizontal direction. Two strain gauges (S1, S2) were symmetrically arranged horizontally in the middle of two sides of columns B and D to measure the deformation of concrete in the horizontal direction in the middle of the column specimen. Two strain gauges (S3, S4) were vertically arranged in the middle of two sides of columns B and D to measure the vertical deformation of concrete in the middle of the column specimen. The detailed layout of the axial compression test is shown in Figure 2.

When formally loading, the 5000 kN electro-hydraulic servo pressure testing machine program was set as a constant speed displacement loading system. The loading rate was always kept at 0.20 mm/min, and the tests were controlled through displacement in stages. Each stage was incremented by 0.30 mm. And then, the tests entered the load-holding state. The test layout is shown in Figure 2.

## 3. Results

### 3.1. Failure Process and Failure Pattern

**Figure 3:**
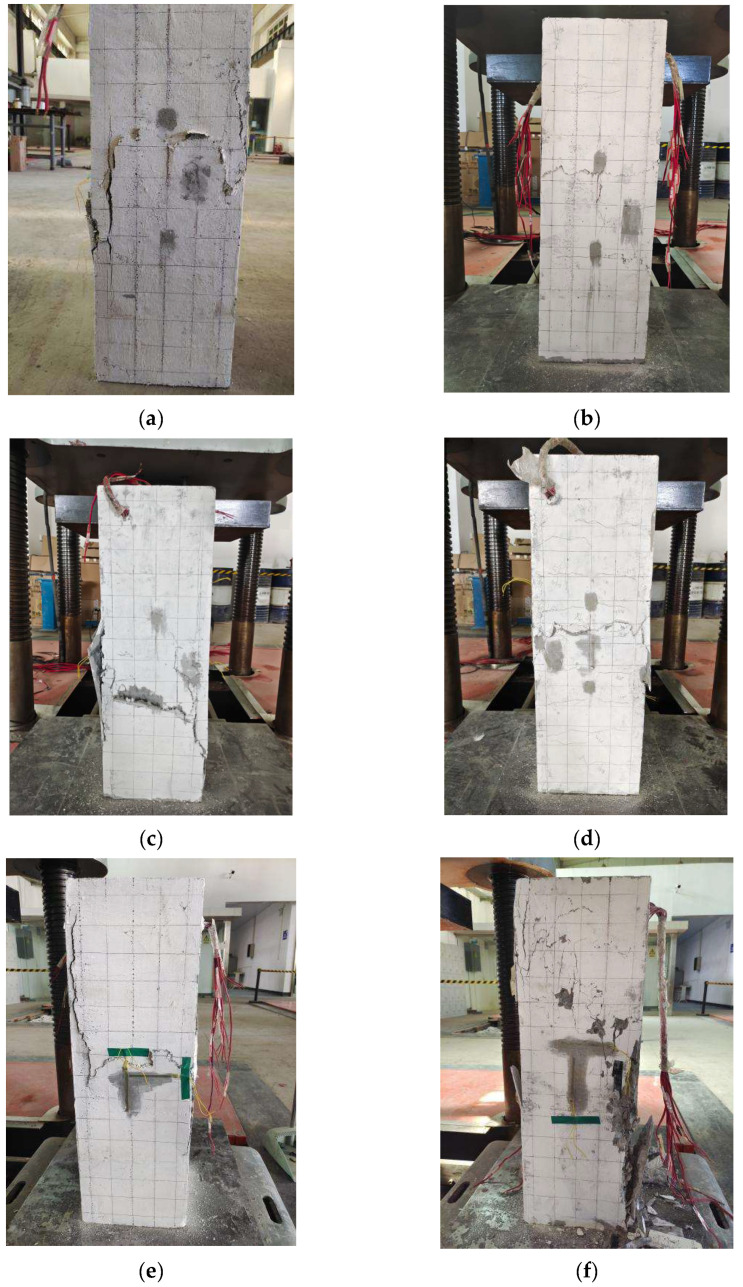

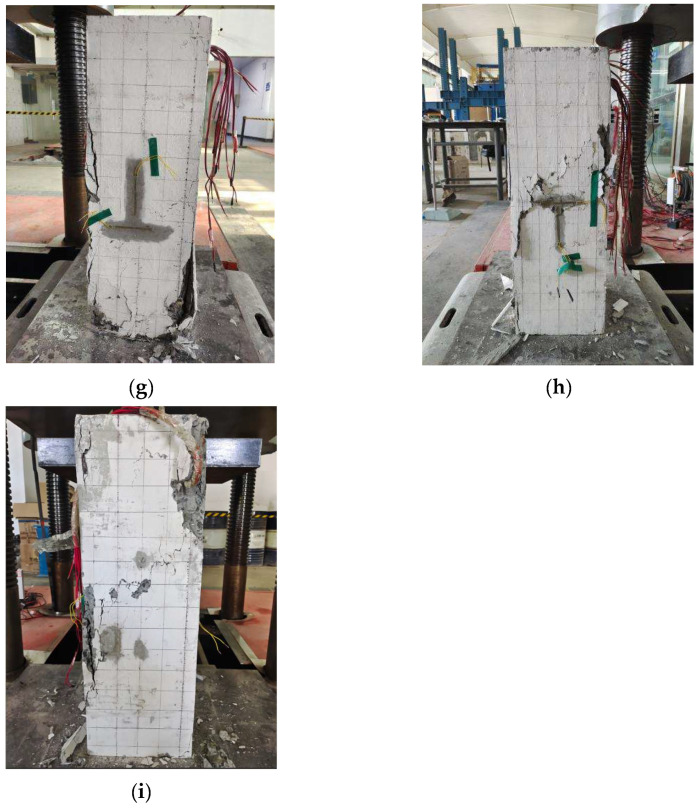
Test piece destruction diagram. (**a**) FC-P-01 overall destruction diagram; (**b**) FC-P-02 overall destruction diagram; (**c**) FC-P-03 overall destruction diagram; (**d**) FC-P-04 overall destruction diagram; (**e**) FC-P-05 overall destruction diagram; (**f**) RC-P-01 overall destruction diagram; (**g**) RC-P-02 overall destruction diagram; (**h**) RC-P-03 overall destruction diagram; (**i**) RC-C-1 overall destruction diagram.

Under the action of axial pressure, the crack development of each specimen in the loading test, and the failure state of RC columns and HPFRCC composite columns were different. The final states are shown in Figure 3.

- For specimen RC-P-01 (Figure 3f), vertical cracks began from the upper column at the load of 449.82 kN. And the cracks appeared and developed vertically and obliquely with the increase in the load. Longitudinal cracks appeared in the lower part of the specimen when the load reached 2550 kN. When the load reached 3204.43 kN, the cracks formed an oblique penetration from the upper to the lower part, and the concrete of the shell began to fall off. At last, the longitudinal crack width of the specimen expanded, the concrete shell at the edges and corners began to peel off in large quantities, the crushing phenomenon occurred at the A side of the specimen, and the reinforced bars were exposed.
- For specimen FC-P-01 (Figure 3a), the specimen showed no surface phenomenon at the beginning. Vertical micro-cracks occurred from the upper of the column at the load of 999.67 kN. The crack in a vertical direction on the right side in the face A developed to the middle part, and then the transverse crack ran through the cross-section at the load of 2640.17 kN. When the peak load was achieved, on face A, the middle part of the specimens was cracked laterally and slightly convex, and the transverse cracks connected with the vertical cracks on both sides of the left and right sides, forming an “H” shape. When the test stopped, the specimen still showed better integrity.
- Compared with FC-P-01, RC-P-01, and RC-C-1, it can be seen that the failure state of RC-C-1 was the most serious, the column body showed obvious brittle failure, and there were four large damages at the corners, resulting in large fragments falling and exposing the stirrup. RC-P-01 was also a brittle failure, but compared with RC-C-1, RC-P-01 has only two damages at the corners and fewer cracks in the column body. Compared with RC-C-1 and RC-P-01, specimen FC-P-01 was in the form of ductile destruction, and only cracks appeared at the edges and corners, and the precast shell maintained good integrity (Figure 3a,f,i).
- Compared with FC-P-01 (*ρ*_v_ was 1.05%), FC-P-02 (*ρ*_v_ was 1.48%), and FC-P-03 (*ρ*_v_ was 0.74%), the damage state of the HPFRCC composite column was more serious with the decrease in the volume stirrup rate. The outer shell of FC-P-03 even appeared to break (Figure 3c). The damage degree in the middle of the precast shell was reduced after increasing the volume stirrup ratio, and the cracks in a vertical direction at the corners of specimens were less. The ductility of composite columns made of reinforced HPFRCC shell was improved, and the development of cracks was also limited, most of which were slender and dense cracks. The overall integrity of FC-P-02 showed the best performance among the three (Figure 3b). Compared with RC-P-01 (*ρ*_v_ was 1.05%), RC-P-02 (*ρ*_v_ was 1.48%), and RC-P-03 (*ρ*_v_ was 0.74%), through the phenomenon analysis, with the increase in the *ρ*_v_, the damage degree of the RC composite column was decreased, and the three columns all showed crushing phenomena at the column foot (Figure 3f–h). Both RC-P-03 and RC-P-01 had large spalling of the outer shell. The outer shell of specimen RC-P-02 was broken at the corner of the column foot, but there was no spalling. Moreover, the width and depth of vertical cracks of the RC composite column decreased with the increase in the stirrup ratio and increasing the volume stirrup ratio could reduce the possibility that the outer shell of the specimen fell at the cross of cracks. In addition, increasing the *ρ*_v_ improved the restraint effect of the precast shell, which was equivalent to improving the deformation ability of the columns.

### 3.2. Load−Deformation Curves

**Figure 4:**
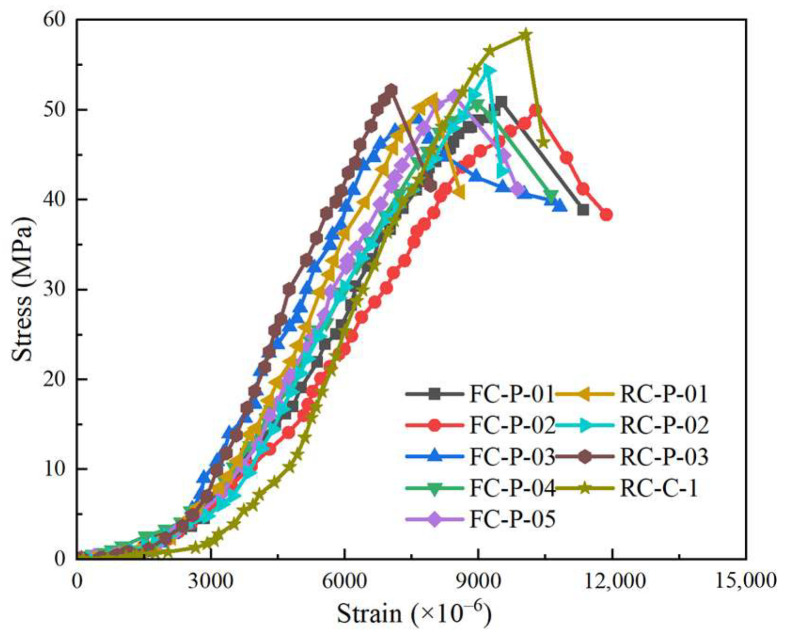
Axial load–vertex displacement curve.

The dimensionless curves of axial load–vertex displacement of the nine columns are presented in Figure 4, and the main key point values of the nine columns are listed in Table 6.

- Compared with specimen RC-P-01, RC-P-02, and RC-P-03, the compressive strain at the yielding point of specimen FC-P-01, FC-P-02, and FC-P-03 increased by 11.25%, 12.49%, and 11.17%, the compressive strain at the peak load point of specimen FC-P-01, FC-P-02, and FC-P-03 increased by 19.43%, 10.86%, and 2.58%.
- Based on Table 6, compared to FC-P-01, the yielding load of FC-P-04 and FC-P-05 increased by 1.54% and 4.10%; the maximum load of FC-P-04 and FC-P-05 increased by 0.73% and 1.40%. The larger the reinforcement ratio of longitudinal reinforcement was, the larger the yield load and peak load of the composite column under axial compression was.
- As seen from Table 6, compared with FC-P-03, the yielding load of FC-P-01 and FC-P-02 increased by 2.42%, 13.09%. The stain at the yielding point of FC-P-01 and FC-P-02 increased by 11.32%, 35.25%. The stain at the maximum load point of FC-P-01 and FC-P-02 increased by 31.75%, 41.31%. The factor of *ρ*_v_ significantly influenced the compressive strain at the characteristic point of the composite column in the axial compression test. Increasing the *ρ*_v_ of the specimens was helpful to its deformation performance. Moreover, the stirrup provided transverse constraint to the composite column, limited its transverse deformation, and was helpful in improving its own compressive bearing capacity.

### 3.3. Load−Strain Curves

#### 3.3.1. Stirrup Strain

**Figure 5:**
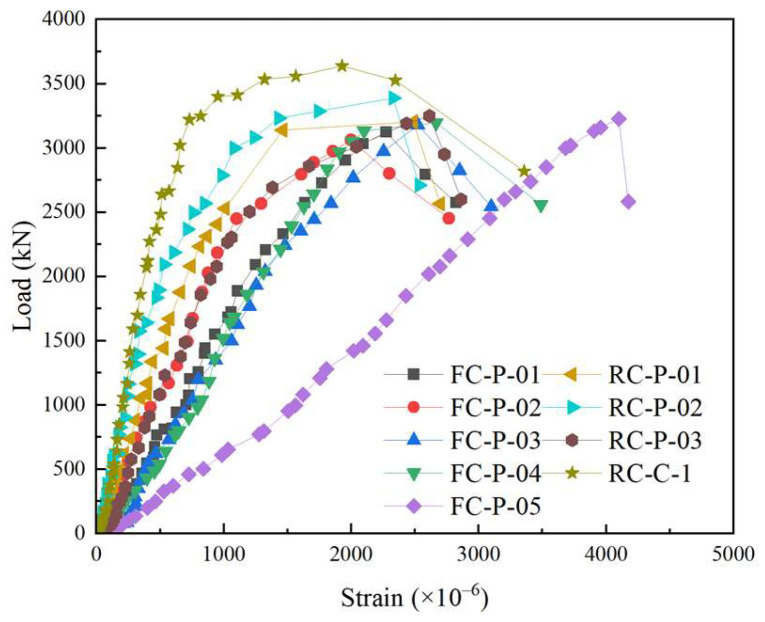
Load–strain curve of stirrups.

The constraint effect on the post-cast core concrete can be reflected in the stirrup strain. The curves are presented in Figure 5, and the stirrup strains are shown in Table 7.

- Initially, in the elastic phase, the strain was linearly related to the axial load. When the load was close to 60–80% of the peak load, the stiffness decreased, the increase rate of the stress also decreased and presented a curve shape. The descending speed of HPFRCC specimens was slower than that of RC specimens. In this stage, the bearing capacity gradually decreased until the specimen failed.
- For the RC-P-01 column and FC-P-01 column, at the initial loading stage of the specimen, the variation trend of the stress–strain curve was roughly similar, with linear growth. In the early loading stage, the stirrup strain grew slowly. And after reaching 80% of the maximum load, the stirrup strain growing rate was rapid. During the test, after the stage of peak load, the specimen RC-P-01 declined faster than the specimen FC-P-01.
- Compared with RC-P-01, RC-P-02, and RC-P-03, the stirrup strain at the peak load point of FC-P-01, FC-P-02, and FC-P-03 reduced by 8.54%, 14.22%, and 3.52%, respectively. As the HPFRCC material had better tensile capacity, the precast shell made of HPFRCC provided additional restraint, thus improving the restraint effect.

#### 3.3.2. Longitudinal Reinforced Bars Strain

**Figure 6:**
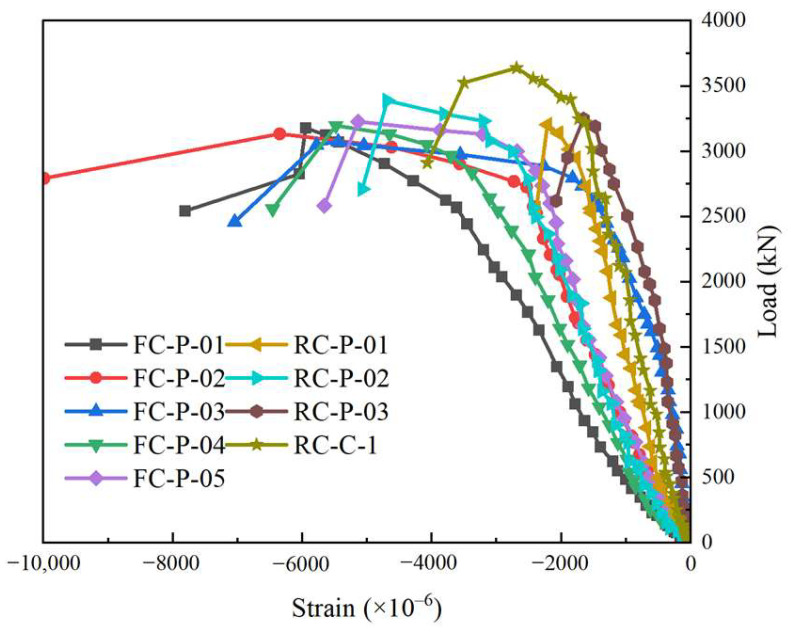
Load–strain curve of longitudinal rebars.

Comparison between HPFRCC specimens and RC specimens (such as FC-P-01 and RC-P-01 specimens), during the initial stage of loading, the strain of both specimens showed a linear growth, and the growth trend of the stress–strain curve also showed a similar relationship. The strain of longitudinal reinforcement was small, and the growth rate was slow. Then, the strain increased rapidly after reaching 80% of the maximum load. The curve rising speed of the RC specimen was faster than that of the HPFRCC specimen during the whole loading test. The stirrup of RC specimens reached yield earlier and peak strain earlier than HPFRCC specimens. As shown in Figure 6, specimens with smaller longitudinal reinforcement ratios (such as FC-P-01, FC-P-04, FC-P-05) showed a faster increase in the curve (from −1.41 to −1.19 and −0.84). After the peak load, the larger the longitudinal reinforcement ratio was, the slower the curve decline rate was. The peak longitudinal reinforcement strain of FC-P-02, FC-P-01, and FC-P-03 decreased successively. Compared with FC-P-01, the value of FC-P-02 increased by 6.74%, while the value of FC-P-03 decreased by 2.84%.

### 3.4. Calculation Model of Axial Compression Capacity of HPFRCC Composite Column

Considering the mechanical properties of HPFRCC material, the force forms of the columns composed of the prefabricated shell of HPFRCC material, and the post-cast concrete were different from those of ordinary concrete. The compressive capacity and deformation of the composite component was one of its important mechanical indexes, through a suitable HPFRCC assembly integral composite column axial bearing capacity model and deformation model, to analyze the composite column axial pressure performance. It had practical significance for its engineering application. Based on the data of this mechanical property test and the existing theory, the axial compressive capacity calculation model of the HPFRCC assembly integral composite column was derived, the peak stress and corresponding strain of HPFRCC columns were analyzed, and the theoretical model of the peak stress of the HPFRCC column, the peak strain of HPFRCC composite column and the axial compressive capacity of the HPFRCC column was proposed.

#### 3.4.1. Material Stress–Strain Relationship

(1) Stress–Strain Relationship of Concrete

**Figure 7:**
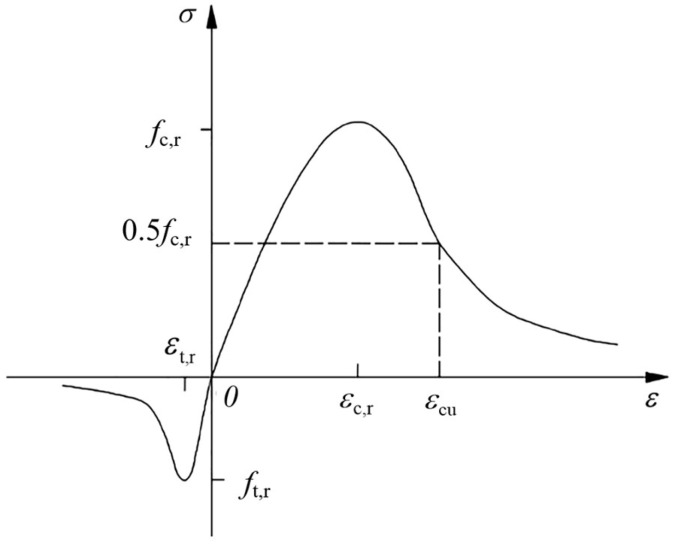
Concrete uniaxial stress–strain curve [12].

According to the model provided in the code [12], the stress–strain constitutive relationship curve of concrete is shown in Figure 7 and can be determined according to the following formula:

(1) $$\sigma =(1-d_{t})E_{c}\varepsilon$$

(2) $$d_{t}=\left\{\begin{array}{ll} 1-\rho_{t}\left[1.2-0.2x^{5}\right] & x\leq 1\\ 1-\frac{\rho_{t}}{\alpha_{t}{\left(x-1\right)}^{1.7}+x} & x>1 \end{array}\right.$$

(3) $$x=\frac{\varepsilon }{\varepsilon_{t,r}}$$

(4) $$\rho_{t}=\frac{f_{t,r}}{E_{c}\varepsilon_{t,r}}$$

where

*α*_t_—parameter values of the descending section of the uniaxial tensile stress–strain curve of concrete are obtained according to the code;

*f*_t,r_—the uniaxial tensile strength of concrete;

*ε*_t,r_—the peak tensile strain of concrete corresponding to the uniaxial tensile strength *f*_t,r_;

*d*_t_—concrete uniaxial tensile damage parameter.

The stress–strain curve of concrete under uniaxial compression is shown in Figure 7, which can be determined according to the following formula:

(5) $$\sigma =(1-d_{c})E_{c}\varepsilon$$

(6) $$d_{c}=\left\{\begin{array}{ll} 1-\frac{{\rho_{c}}^{n}}{n-1+x^{n}} & x\leq 1\\ 1-\frac{\rho_{c}}{\alpha_{c}{\left(x-1\right)}^{2}+x} & x>1 \end{array}\right.$$

(7) $$\rho_{c}=\frac{f_{c,r}}{E_{c}\varepsilon_{c,r}}$$

(8) $$n=\frac{E_{c}\varepsilon_{c,r}}{E_{c}\varepsilon_{c,r}-f_{c,r}}$$

(9) $$x=\frac{\varepsilon }{\varepsilon_{c,r}}$$

where

*α*_c_—the parameter value of the descending section of the stress–strain curve of concrete under uniaxial compression;

*f*_c,r_—the uniaxial compressive strength of concrete;

*ε*_c,r_—the peak tensile strain of concrete corresponding to the uniaxial tensile strength *f*_c,r_;

*d*_c_—concrete uniaxial compression damage parameter.

(2) Constitutive model of reinforcement

**Figure 8:**
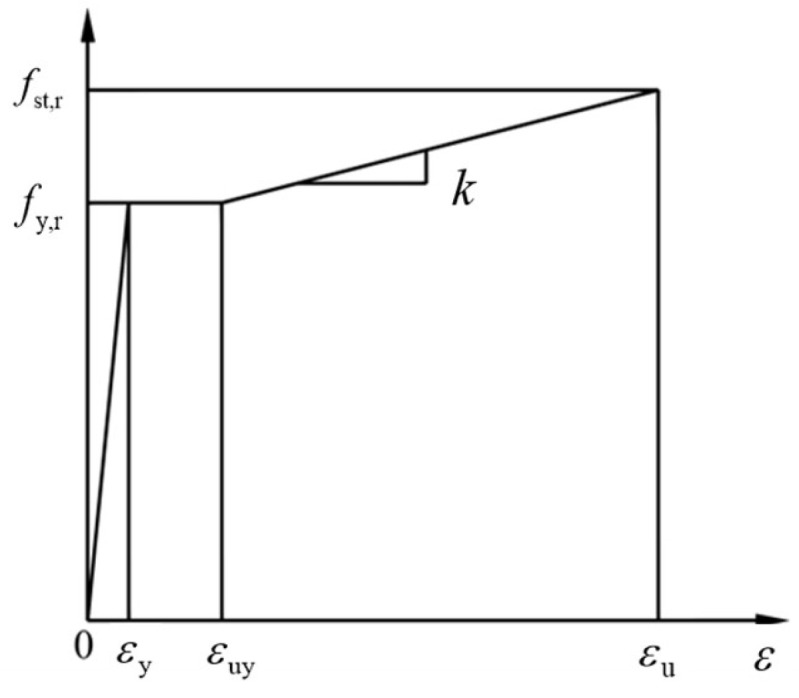
Uniaxial stress–strain curve of steel bar.

According to the stress–strain curve of reinforcement in the specification [12] (Figure 8), it can be determined according to the following provisions.

(10) $$\sigma_{s}=\left\{\begin{array}{ll} E_{s}\varepsilon_{s} & \varepsilon_{s}\leq \varepsilon_{y}\\ f_{y,r} & \varepsilon_{y}<\varepsilon_{s}\leq \varepsilon_{\mathrm{uy}}\\ f_{y,r}+k(\varepsilon_{s}-\varepsilon_{\mathrm{uy}}) & \varepsilon_{\mathrm{uy}}<\varepsilon_{s}\leq \varepsilon_{u}\\ 0 & \varepsilon_{s}>\varepsilon_{u} \end{array}\right.$$

where

*E*_s_—elastic modulus of reinforcement;

*σ*_s_—reinforcement stress;

*ε*_s_—reinforcement strain;

*f*_y,r_—the representative value of the yield strength of the steel bar, its value according to the actual structural analysis needs to take *f*_y_*, f*_yk_ and *g*_ym_ (the design value of the yield strength of the steel bar, standard value or average value);

*ε*_y_—the yield strain of the steel bar corresponding to *f*_y,r_, preferably *f*_y,r_/*E*_s_;

*ε*_uy_—starting strain of steel bar hardening;

*ε*_u_—the peak strain of the steel bar corresponding to *f*_st,r_;

*k*—the slope of the hardening section of the rebar, *k* = (*f*_st,r_ − *f*_y,r_)/(*ε*_u_ − *ε*_uy_).

(3) Constitutive model of HPFRCC

Based on the stress–strain relationship of the HPFRCC and referring to the uniaxial compression constitutive equation proposed by Li [13]. The dimensionless formula is expressed as follows:

(11) $$y=\left\{\begin{array}{ll} \frac{Ax-x^{2}}{1+(A-2)x} & 0\leq x<1\\ \frac{A_{1}x}{1+(A_{1}-2)+x^{2}} & x\geq 1 \end{array}\right.$$

where

*x* = *ε*/*ε*_0_ (*ε* and *ε*_0_ are the strain and peak strain of HPFRCC);

*y* = *σ*/*σ*_0_ (*σ* and *σ*_0_ are, respectively, the stress and peak stress of HPFRCC);

*A* is 1.101;

*A*_1_ = (0.0234 + 0.1577*V*_f_)(0.0016*f*_c_^2^ − 0.2315*f*_c_ + 8.4633);

*V*_f_—fiber volume content of HPFRCC;

*f*_c_—axial compressive strength of HPFRCC.

**Figure 9:**
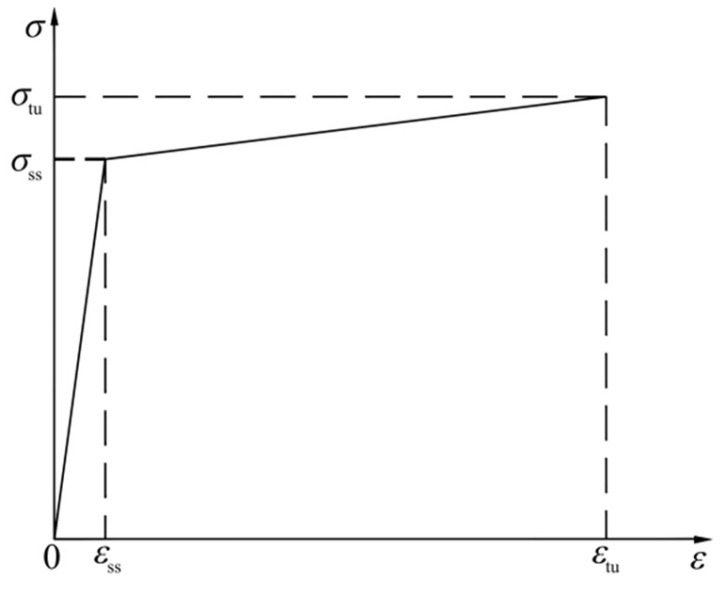
HPFRCC uniaxial tension bilinear model.

Based on the characteristics of HPFRCC tensile strain hardening, domestic and foreign scholars adopted the bilinear model [14,15], as shown in Figure 9, the first stage is before reaching the cracking stress, the stress–strain curve shows linear growth, the second stage is the tensile strain hardening stage, the stress–strain curve also shows linear growth, the tension constitutive model of HPFRCC adopts the following:

(12) $$\sigma (\varepsilon )=\left\{\begin{array}{ll} E_{c}\varepsilon & \varepsilon \leq \sigma_{\mathrm{ss}}/E\\ \sigma i+E_{ie}\sigma & \varepsilon >\sigma_{\mathrm{ss}}/E \end{array}\right.$$

(13) $$E_{ie}=\frac{\sigma_{\mathrm{tu}}-\sigma_{\mathrm{ss}}}{\sigma_{\mathrm{tu}}-\sigma_{\mathrm{ss}}/E_{c}}$$

(14) $$\sigma_{i}=\sigma_{\mathrm{ss}}\left(1-\frac{E_{ie}}{E_{c}}\right)$$

where

*E*_c_ = *f*_cu_^0.596^ × 10^3^ (MPa);

*σ*_ss_—stable cracking load stress, *σ*_ss_ = 0.896*σ*_tu_;

*σ*_tu_—ultimate tensile strength, *σ*_tu_ = 3.683*f*_cu_^0.714^.

#### 3.4.2. Constraint Coefficient

In this mechanical properties test of the HPFRCC assembled monolithic composite column, the axial load was borne by the HPFRCC outer shell, post-cast core concrete, longitudinal reinforcement, stirrup, and HPFRCC layer. During the test process, the HPFRCC outer shell, the after-cast core concrete and the longitudinal reinforcement maintained good cooperative work, showing good adhesion and no slip and separation phenomenon.

Although the superposition method is used to calculate the axial compressive capacity of the HPFRCC precast monolithic composite column as a whole, the bearing capacity of the hooped reinforcement and HPFRCC confined layer cannot be calculated using only the simple superposition method. Therefore, the influencing factor of the constraint coefficient is considered to obtain the corresponding bearing capacity relatively accurately.

**Figure 10:**
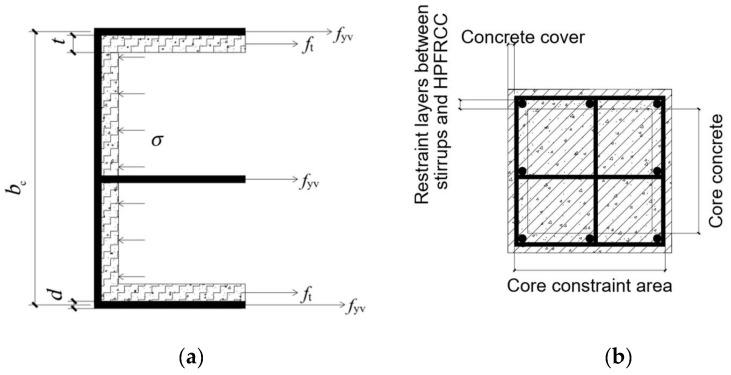
Stress distribution of stirrups and HPFRCC. (**a**) Structure diagram of superimposed columns. (**b**) Stress distribution diagram.

The constraint stress of the HPFRCC composite column consists of two parts: the first part is the transverse constraint of the stirrup, and the second part is the constraint effect of the HPFRCC layer, as shown in Figure 10:

Stirrups lateral binding

(1) The influence of stirrup characteristic value.

The stirrup characteristic value was proportional to its transverse binding force. It was assumed that after the stirrup yields, the transverse constraint stress of the stirrup to the HPFRCC layer was uniformly distributed along the direction of the stirrup. For cross-type composite stirrup, the transverse constraint stress of the stirrup can be obtained from Figure 10 as follows:

(15) $$\sigma_{h}=\sigma_{x,\mathrm{outside}}+\sigma_{x,\mathrm{inside}}=\frac{3f_{\mathrm{yv}}A_{\mathrm{sv}}}{b_{c}s}$$

The volume stirrup ratio of the cruciform stirrup is as follows:

(16) $$\rho_{v,\mathrm{outside}}=\frac{2(b_{c}+d_{c})A_{\mathrm{sv},\mathrm{outside}}}{A_{\mathrm{cor}}s}$$

(17) $$\rho_{v,\mathrm{inside}}=\frac{\left(b_{c}+d_{c}\right)A_{\mathrm{sv},\mathrm{inside}}}{A_{\mathrm{cor}}s}$$

(18) $$\lambda_{v}=\rho_{v}\frac{f_{\mathrm{yv}}}{f_{c}}$$

Bring Formulas (16)–(18) into Formula (15) and *b*_c_ = *d*_c_ to achieve Formula (19)

(19) $$\sigma_{h}=(\frac{\lambda_{v,\mathrm{outside}}}{2}+\frac{\lambda_{v,\mathrm{inside}}}{2})f_{c}$$

where

*σ*_h_—the transverse binding force of the cross-type composite stirrup;

*σ_x_*_,outside_—the lateral binding force of the outer stirrup;

*σ_x_*_,inside_—the transverse force of the inner stirrup;

*f*_yv_—yield stress of the stirrup;

*A*_sv_—the cross-sectional area of the outer stirrup;

*b*_c_, *d*_c_—the length and width of the outer stirrup;

*s*—the spacing between the stirrups; 

*ρ*_v,outside_—the ratio of the outer stirrup volume;

*ρ*_v,inside_—inner stirrup volume ratio;

*A*_sv,inside_—cross-sectional area of inner stirrup;

*A*_sv,outside_—cross-sectional area of the outer stirrup;

*A*_cor_—the area enclosed by the outer stirrup;

*λ*_v_—stirrup characteristic value;

*f*_c_—the axial compressive strength of HPRCC material;

*λ*_v,outside_—the stirrup characteristic value of the external stirrup of the cross-type conforming to the stirrup;

*λ*_v,inside_—the stirrup characteristic value of the inner stirrup of the stirrup conforming to the stirrup.

(2) Influence of stirrup form

The greatest constraint effect exists at the welding and four corners of the composite stirrup and longitudinal reinforcement, and the weakest constraint is in the middle of the two adjacent longitudinal reinforcements. Through the research of existing scholars (the ratio of effective restraint area and core area [16], the influence of stirrup form on the constraint effect can be calculated as follows:

(20) $$A_{e,n}=b_{c}d_{c}-\sum_{i=1}^{n}\frac{{w_{ix}}^{2}+{w_{iy}}^{2}}{6}$$

The influence coefficient of the stirrup [17] is as follows:

(21) $$\alpha_{n}=1-\sum_{i=1}^{n}\frac{{w_{ix}}^{2}+{w_{iy}}^{2}}{6b_{c}d_{c}}$$

where

*A*_e,n_—the cross-section effective restraint area of the outer stirrup;

*w_ix_*—the distance between two adjacent longitudinal bars on one side of the outer stirrup;

*w_iy_*—the spacing of two adjacent longitudinal bars on the other side of the outer stirrup.

(3) The effect of stirrup spacing

The place with the smallest effective restraint area is in the middle of the adjacent stirrup. Due to the concept of safety design and the research of existing scholars [18], it is selected as the control interface, and the effective restraint line along the longitudinal column is approximately equivalent to a semicircle, and the vertex of the semicircle is 1/2 of the stirrup spacing, then *A*_es_ is calculated as follows:

(22) $$A_{\mathrm{es}}=(b_{c}-\frac{s^{\prime }}{2})(d_{c}-\frac{s^{\prime }}{2})$$

Then, the influence coefficient of stirrup spacings is as follows:

(23) $$\alpha_{s}=(1-\frac{s^{\prime }}{2b_{c}})(1-\frac{s^{\prime }}{2d_{c}})$$

where

*A*_es_—the effective restraint area of the control section of the outer stirrup;

*s′*—net stirrup spacing.

The three influencing factors of stirrup characteristic value, stirrup form, and stirrup distance are combined, and the three factors are taken into consideration. According to Equations (18), (19), (21) and (23), the confining stress of the stirrup is as follows:

(24) $$\sigma_{h}=(\frac{\alpha_{n}\alpha_{s}\lambda_{v,\mathrm{outside}}}{2}+\frac{\lambda_{v,\mathrm{inside}}}{2})f_{c}$$

Refer to foreign scholars to introduce dimensionless coefficient *I*_e_:

(25) $$I_{e}=\frac{\sigma_{h}}{f_{c}}=\frac{\alpha_{n}\alpha_{s}\lambda_{v,\mathrm{outside}}}{2}+\frac{\lambda_{v,\mathrm{inside}}}{2}$$

where

*I*_e_—effective constraint coefficient of the cross-type composite stirrup.

Horizontal constraint of HPFRCC layer

(26) $$\sigma_{H}=\frac{2f_{t}t}{b_{c}}$$

By the same token, dimensionless coefficient *I*_h_ is introduced as follows:

(27) $$I_{h}=\frac{\sigma_{H}}{f_{c}}=\frac{2f_{t}t}{b_{c}f_{c}}$$

Therefore, the comprehensive binding force *σ* and effective constraint coefficient *I_m_* of HPFRCC composite columns are expressed as the following formula:

(28) $$\sigma =\sigma_{h}+\sigma_{H}=(\frac{\alpha_{n}\alpha_{s}\lambda_{v,\mathrm{outside}}}{2}+\frac{\lambda_{v,\mathrm{inside}}}{2})f_{c}+\frac{2f_{t}t}{b_{c}}$$

(29) $$I_{m}=\frac{\sigma }{f_{c}}=\frac{\sigma_{h}+\sigma_{H}}{f_{c}}=I_{e}+I_{h}=\frac{\alpha_{n}\alpha_{s}\lambda_{v,\mathrm{outside}}}{2}+\frac{\lambda_{v,\mathrm{inside}}}{2}+\frac{2f_{t}t}{b_{c}f_{c}}$$

where

*I*_h_—effective constraint coefficient of HPFRCC material protective layer;

*f*_t_—HPFRCC material tensile stress;

*I*_m_—the effective restraint factor of the stirrup and HPFRCC protective layer is considered.

#### 3.4.3. Peak Stress Calculated Model

The cover depth of all HPFRCC composite column specimens in this paper is 10 mm, so its influence should be considered in the calculation. Therefore, in this paper, the bearing capacity of the specimen is regarded as provided by the core constraint area, longitudinal reinforcement, and protective layer. The calculation expression is as follows:

(30) $$N_{h}=N_{c}+N_{z}+N_{m}$$

where

*N*_c_ = *σ*_cc_*A*_cor_;

*N*_z_ = *f*_y_*A*_s_;

*N*_m_ = *f*_c_*A*_cov_;

*N*_h_—peak load during axial compression;

*N*_c_—the peak load on the core confinement area;

*N*_z_—the peak load provided by the longitudinal bar;

*N*_m_—the peak load provided by the protective layer;

*σ*_cc_—peak stress in the core confinement zone;

*A*_cor_—area of the core confinement zone;

*f*_y_—yield strength of longitudinal bar;

*A*_s_—cross-sectional area of all longitudinal bars;

*f*_c_—the axial compressive strength of HPFRCC material;

*A*_cov_—the cross-sectional area of the protective layer.

Therefore, the formula for calculating the peak stress can be obtained:

(31) $$\sigma_{\mathrm{cc}}=\frac{N_{h}-f_{y}A_{s}-f_{c}A_{\mathrm{cov}}}{A_{\mathrm{cor}}}$$

The peak stresses of nine columns were calculated using the Formula (31) and compared with the experimental compressive strength of the concrete axis. The calculation results are listed in Table 8 below.

**Figure 11:**
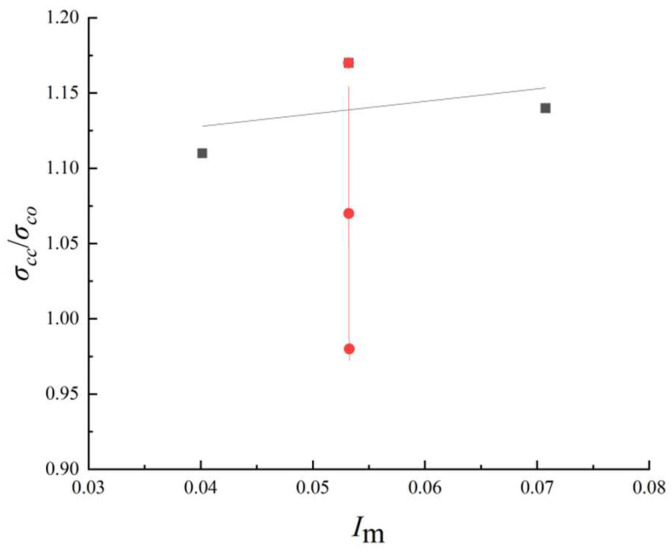
Relationship between *σ*_cc_/*σ*_co_ and *I*_m_.

For the specimens made of HPFRCC material, *ρ*_v_ and *ρ*_t_ are used as variables. The effective constraint coefficient *I*_m_ represents the influence of the constraint stress change in the stirrup and HPFRCC confined layer on the column mechanics performance. Moreover, both the outer shell and the stirrup of HPFRCC exert lateral constraints on the core area, playing the same role. Figure 11 shows the scatter relationship between *σ*_cc_/*σ*_co_ and effective constraint index *I*_m_. The changes of *σ*_cc_/*σ*_co_ and *I*_m_ have a linear positive correlation with *ρ*_v_ and *ρ*_t_, which increases with the reinforcement ratio of longitudinal reinforcement.

HPFRCC specimens numbered FC-P-01, FC-P-04, and FC-P-05 declined successively in the figure, but the increase in effective constraint coefficient *I*_m_ was small, indicating that the factor *ρ*_t_ had little influence on the effective constraint index, and the reinforcement ratio of longitudinal reinforcement increased from 1.45% of FC-P-01 to 1.97% of FC-P-04 and 2.57% of FC-P-05. The peak stress in the core confinement zone decreased successively, which indicated to a certain extent that the increase in the reinforcement ratio can share more axial load for the core zone, so the peak stress in the confinement zone decreased accordingly. The factor *ρ*_v_ of specimens FC-P-01, FC-P-02, and FC-P-03 increased successively, and the corresponding *I*_m_ also increased. It indicated that the factor *ρ*_v_ had a significant impact on the effective confinement index, but the peak stress has little difference, indicating that the factor *ρ*_v_ has little effect on the peak stress in the core confinement region.

The core part of the HPFRCC specimens is subject to the lateral constraint of the HPFRCC prefabricated shell, so the theoretical value can be calculated using the formula concerning the research of scholars [19]. The formula is as follows:

(32) $$\frac{\sigma_{\mathrm{cc}}}{\sigma_{\mathrm{co}}}=1+\alpha \frac{\sigma_{3}}{\sigma_{\mathrm{co}}}$$

where 

*α*—effective coefficient of lateral restraint;

*σ*_co_—the peak stress (axial compressive strength) when unconstrained;

*σ*_cc_—peak stress when restrained.

As can be seen from the analysis of Figure 11, the peak stress of the HPFRCC composite column shows a linear growth relationship with the constraint layer composed of the stirrup and HPFRCC layer. Therefore, combined with Formula (32), it is converted into the relationship between the HPFRCC composite column and constraint coefficient as follows:

(33) $$\frac{\sigma_{\mathrm{cc}}}{\sigma_{\mathrm{co}}}=1+k\frac{\sigma }{\sigma_{\mathrm{co}}}$$

Substitute Formula (29) into Formula (33) to achieve the new formula as follows:

(34) $$\frac{\sigma_{\mathrm{cc}(m)}}{\sigma_{\mathrm{co}(m)}}=1+k_{m}I_{m}$$

By combining the Formula (34) and the linear relationship between *σ*_cc_/*σ*_co_ and *I*_m_, the HPFRCC composite column, *k*_m_ = 5.182 can be obtained by linear regression, is as follows:

(35) $$\frac{\sigma_{\mathrm{cc}(m)}}{\sigma_{\mathrm{co}(m)}}=1+5.182I_{m}$$

where

*σ*_cc(m)_—the peak stress of σ-HPFRCC composite column;

*σ*_co(m)_—peak stress when the concrete is unrestrained (*f_c_*);

*I*_m_—effective confinement factor.

#### 3.4.4. Bearing Capacity Calculated Model

In the axial compression test, the longitudinal reinforcement and the hoop had good adhesion with the HPFRCC outer shell and the core concrete, and the deformation was coordinated during the test. The protective layer of the HPFRCC composite column only had dense micro-cracks on the surface, no slippage or other phenomena, and the composite column showed good integrity. Therefore, this paper believes that the bearing capacity is provided by the core concrete, longitudinal reinforcement, hoop, and HPFRCC layer of the restraint area and the protective layer. Thus, the HPFRCC composite column specimen bearing capacity expression could be obtained as follows:

(36) $$N_{H}=N_{c}+N_{z}+N_{s}+N_{m}$$

(37) $$N_{H}=\sigma_{\mathrm{co}(m)}\times A_{\mathrm{cor}}+f_{y}A_{S}+(1+5.182I_{m})\times f_{c}\times A_{\cos}+f_{c}A_{\mathrm{cov}}$$

where

*N*_H_—calculated value of axial compressive capacity of HPFRCC composite column specimens;

*N*_c_—the bearing capacity of core concrete;

*N*_z_—the bearing capacity provided by the longitudinal reinforcement;

*N*_s_—the bearing capacity provided by the stirrup and HPFRCC layer;

*N*_m_—the bearing capacity provided by the protective layer;

*σ*_co(m)_—the axial compressive strength of the core concrete;

*A*_cor_—section area of the core concrete;

*f*_c_—axial compressive strength of HPFRCC;

*A*_cos_—the section area of HPFRCC in the confinement zone;

*f*_y_—the yield strength of the longitudinal bar;

*A*_s_—the cross-sectional area of all the longitudinal bars;

*A*_cov_—the cross-sectional area of the protective layer.

## 4. Discussions

### 4.1. Analysis of Experimental Results

The experimental results present the improved performance of HPFRCC composite columns compared with RC composite columns, particularly in terms of deformation capacity, crack control, and structural integrity.

- The results demonstrate that the incorporation of HPFRCC materials enhances the deformation performance, structural integrity, and load-bearing capacity of composite columns compared with RC composite columns. Through comparisons between RC-P-01 and FC-P-01, RC-P-02 and FC-P-03, and RC-P-03 and FC-P-03, it is evident that the HPFRCC material plays a critical role in improving the ductility and overall behavior of the composite columns. Unlike RC columns, which exhibited severe issues such as the detachment of the precast shell and the formation of large-scale cracks, the HPFRCC composite columns maintained their structural integrity even after failure, exhibiting a more ductile failure mode. During the testing process, the HPFRCC material exhibited strain-hardening behavior when the prefabricated shell cracked. The fibers within the HPFRCC acted as bridges at the crack locations, effectively transferring stress and controlling further crack propagation. This mechanism not only minimized the development of localized large cracks but also prevented the formation of long and wide cracks, which were commonly observed in the RC composite columns. Instead, the HPFRCC composite columns developed a dense network of fine cracks on their surfaces, allowing them to maintain overall integrity post-failure.
- Once peak stress was reached, the RC composite columns experienced a rapid drop in reinforcement bar stress and a significant reduction in deformation resistance, but the HPFRCC composite columns achieved a larger strain at the peak load point. Moreover, when the HPFRCC specimens reached their peak stress, they showed a larger deformation capacity, as evidenced by the gradual decline in the stress–strain curve after peak stress. This behavior highlights the superior energy absorption and ductility, which are critical for structures subjected to high stress or seismic loads.
- The HPFRCC shell provides enhanced confinement of the post-cast concrete, delaying the yielding of longitudinal bars and stirrup. This delay contributed to the improved deformation performance and load-bearing capacity of the composite columns. This constraint is due to the strain-hardening properties of the HPFRCC material, which restrain lateral expansion and distribute stress effectively. The material’s exceptional tensile deformation performance also allowed the precast shell to withstand greater circumferential expansion, maintaining structural integrity under large deformation. These properties make the HPFRCC composite column ideal for seismic-resistant structures. In the future, it is necessary to conduct further research on the seismic performance of HPFRCC composite columns and provide a basis for its application in engineering structures.

HPFRCC composite columns present apparent advantages over RC composite columns, including enhanced crack control, maintaining structural integrity, good deformability, and higher load-bearing capacity, due to the strain-hardening and fiber-bridging properties of HPFRCC. The ability of the HPFRCC composite column to withstand extreme loads and deformations positions makes it a potential application in high-performance construction projects and structures with seismic resilience requirements. Further research on their seismic performance and long-term behavior will support their broader adoption in engineering practice.

### 4.2. Evaluation of Peak Stress Calculated Model

The peak stresses of the composite columns were calculated using the Formula (31) and compared with the experimental results. The calculation results are listed in Table 8.

Based on the parameters of HPFRCC composite column specimens and the above Formula (35), the theoretical values of the peak stress were calculated, as shown in Table 9. For specimens FC-P-01~FC-P-05, the ratios of the calculated value *σ*_cc(m)_ to the experimental value *σ*_test_ were 0.958, 1.041, 0.938, 0.952, and 0.944. The average value was 0.967, and the sample variance was 0.0018, indicating that the established theoretical model exhibited high consistency with the experimental results.

When calculating the constraint effect factor, it was assumed that the constraint factor of the HPFRCC precast shell increases with the increase in the stirrup ratio. However, there may be some deviations in the actual test. Therefore, for specimen FC-P-02, the calculated constraint factor tends to be larger due to its higher volumetric stirrup ratio, resulting in a slightly higher calculated value than the experimental value. Given the limited experimental data, the reliability of this formula requires further validation and refinement through additional experimental results.

### 4.3. Assessment of Bearing Capacity Calculated Model

The maximum bearing capacity is calculated by Equation (37). The experimental and theoretical calculated values of axial compressive capacity are listed in Table 10. The ratios of theoretical values to experimental values for specimen FC-P-01~FC-P-05 were 0.96, 0.99, 098, 0.99, and 1.08, respectively. The average value of *N*_H_/*N*_t_ is 0.999, and the sample variance is 0.0022. The theoretical model calculated values exhibited a high degree of consistency with the experimental values.

Based on theoretical knowledge, the bearing capacity calculation formula for this paper is established. The calculated results from the theoretical model based on this formula show good agreement with the experimental values. However, the theoretical analysis did not consider the bond-slip of the specimen. The run-in stage between the specimen and the equipment in the preliminary stage of the test was not considered in the theoretical calculation. The initial damage of the specimen was not considered in the calculation, which had an impact on the stiffness and the influence on the deformation was not considered. Due to the limited test data, the accuracy of this formula still requires further validation and refinement through subsequent experimental studies.

## 5. Conclusions

In this paper, the axial compressive properties of HPFRCC monolithic composite columns were studied. During the mechanical properties experiment, five HPFRCC composite columns, three RC composite columns, and one whole cast column were tested, and the effects of prefabricated shell materials, the volume, and the reinforcement ratio of composite columns were compared and analyzed. Conclusions are listed as follows:

- HPFRCC prefabricated composite column showed good axial compression performance. Using HPFRCC material as a prefabricated shell can significantly enhance the column deformability. The compressive strain at the yielding point of the HPFRCC specimens was 11.59% higher than the average value of the RC specimens, and the average value of the peak compressive strain was 10.92%. The HPFRCC shell could provide an additional restraint effect to the specimen. The damage degree of the column decreased to a certain extent, avoiding brittle failure. Then, the deformability and integrity of the columns were enhanced.
- According to the analysis of the experimental results, the factor *ρ*_v_ played a great influence on the deformability of the column. Compared with FC-P-01 (*ρ*_v_ was 1.05%), FC-P-02 (*ρ*_v_ was 1.48%) and FC-P-03 (*ρ*_v_ was 0.74%), based on FC-P-01, the compressive strain at the yielding point of the specimen FC-P-02 increased by 21.63%. The compressive strain at the yielding point of the specimen FC-P-03 decreased by 11.20%.
- Increasing the *ρ*_t_ of longitudinal reinforced bars could enhance the axial compressive capacity. Among HPFRCC specimens, based on the FC-P-01 specimen, the maximum load of the FC-P-04 specimen increased by 0.57%, while that of FC-P-05 increased by 1.49%.
- Based on theoretical analysis, the peak stress model, peak strain model, and bearing capacity calculation model of HPFRCC assembled monolithic composite column was established. The average value of *N*_H_/*N*_t_ is 0.999, and the sample variance is 0.0022. The established peak stress model, peak strain model, and theoretical calculation formula of bearing capacity of HPFRCC assembled monolithic composite column were feasible.

Due to the limited time and experimental data, the above models need to be validated with more experimental data and further revisions.

## Figures and Tables

**Table 1:** Specimen design parameters.

Test Piece	Column Length (mm)	Cross-Section (mm × mm)	Stirrup Spacing (mm)	Column Reinforcement	
				Longitudinal Bars	Stirrups
FC-P-01	750	250 × 250	70	8 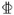 12	*ϕ* 6@70
FC-P-02	750	250 × 250	50	8 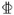 12	*ϕ* 6@50
FC-P-03	750	250 × 250	100	8 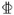 12	*ϕ* 6@100
FC-P-04	750	250 × 250	70	8 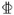 14	*ϕ* 6@70
FC-P-05	750	250 × 250	70	8 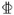 16	*ϕ* 6@70
RC-P-01	750	250 × 250	70	8 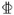 12	*ϕ* 6@70
RC-P-02	750	250 × 250	50	8 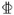 12	*ϕ* 6@50
RC-P-03	750	250 × 250	100	8 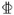 12	*ϕ* 6@100
RC-C-1	750	250 × 250	70	8 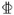 12	*ϕ* 6@70

**Table 2:** HPFRCC mix ratio.

Cement (g/L)	Fly Ash (g/L)	Quartz Sand (g/L)	PVA Fiber (g/L)	Water Reducer (g/L)	Water (g/L)
630	630	406	20	15	436

**Table 3:** Various parameters of PVA fiber.

Fiber Name	Length (mm)	Diameter (µm)	Tensile Strength (MPa)	Elastic Modulus (GPa)	Elongation (%)
PVA	12	40	1560	41	6.5

**Table 4:** Cubic test block compression and tensile test results.

Material Type	Cube Compressive Strength *f*_cu_ (MPa)	Prismatic Compressive Strength *f*_c_ (MPa)	Tensile Strength *f*_t_ (MPa)
HPFRCC	49.9	43.2	5.2
RC	47.2	38.2	3.4

**Table 5:** Average value of steel bar tensile test.

Types of Rebar	Diameter of Rebar (mm)	Yield Strength (MPa)	Ultimate Strength (MPa)
HPB300	6	355	506
HRB400	12	463	642
HRB400	14	442	614
HRB400	16	532	698

**Table 6:** Load and deformation of the characteristic points for specimens.

Number	Yielding Point		Maximum Load Point	
	Load (kN)	Deformation (10^−6^)	Load (kN)	Deformation (10^−6^)
FC-P-01	2630.52	7780.28	3182.55	9519.45
FC-P-02	2904.47	9452.34	3143.63	10,208.67
FC-P-03	2568.13	6988.56	3074.38	7224.52
FC-P-04	2670.92	7416.34	3197.91	8962.67
FC-P-05	2738.34	7280.75	3227.32	8465.33
RC-P-01	2711.88	6993.33	3204.43	7970.67
RC-P-02	2869.62	8402.61	3399.28	9208.76
RC-P-03	2632.45	6286.47	3260.43	7042.87
RC-C-1	2989.32	7954.67	3649.28	10,058.67

**Table 7:** Stirrup strain at the characteristic point of columns.

Number	Peak Loads (kN)	Stirrup Strain (10^−6^)	Yield Load (kN)
FC-P-01	3182.55	2274.07	2630.52
FC-P-02	3143.63	1997.58	2914.47
FC-P-03	3074.38	2522.86	2568.13
FC-P-04	3197.91	2666.80	2670.92
FC-P-05	3227.32	4099.93	2738.34
RC-P-01	3204.43	2486.48	2711.88
RC-P-02	3399.28	2328.51	2869.62
RC-P-03	3260.43	2614.36	2632.45
RC-C-1	3649.28	1927.56	2989.32

**Table 8:** Peak stress test results of HPFRCC/RC composite columns.

Test Piece Number	*σ*_co_ (MPa)	*σ*_cc_ (MPa)	*σ*_cc_/*σ*_co_
FC-P-01	38.2	44.90	1.18
FC-P-02	38.2	43.84	1.15
FC-P-03	38.2	42.71	1.12
FC-P-04	38.2	42.30	1.11
FC-P-05	38.2	37.35	0.98
RC-P-01	38.2	45.53	1.19
RC-P-02	38.2	49.22	1.29
RC-P-03	38.2	46.59	1.22
RC-C-1	38.2	53.94	1.41

Note: *σ*_co_ is the compressive strength of the concrete axis.

**Table 9:** Peak stress of HPFRCC composite columns.

Test Piece Number	*σ*_cc(m)_ (MPa)	*σ*_test_ (MPa)	*σ*_cc(m)_/*σ*_test_
FC-P-01	48.730	50.877	0.958
FC-P-02	52.206	50.141	1.041
FC-P-03	46.146	49.187	0.938
FC-P-04	48.735	51.166	0.952
FC-P-05	48.744	51.653	0.944

**Table 10:** Axial compression bearing capacity of HPFRCC composite columns.

Test Piece Number	*N* (kN)		*N*_H_/*N*_t_
	Calculated Value (*N*_H_)	Test Value (*N*_t_)	
FC-P-01	3045.17	3182.55	0.96
FC-P-02	3106.61	3143.63	0.99
FC-P-03	2999.48	3074.24	0.98
FC-P-04	3170.60	3197.91	0.99
FC-P-05	3481.99	3227.32	1.08

Note: *N*_t_ is the trial value; *N*_H_ is the theoretical calculated value.

## Data Availability

The original contributions presented in the study are included in the article, further inquiries can be directed to the corresponding author.
